# Identification of the Potential Gene Regulatory Networks and Therapeutics in Aged Mice With Postoperative Neurocognitive Disorder

**DOI:** 10.3389/fnins.2021.689188

**Published:** 2021-06-24

**Authors:** Wensi Wu, Yongpai Peng, Jiaxin Zhou, Xiaojun Zhang, Lin Cao, Wei-jye Lin, Yanan Lu, Jing Wen, Zhi Wang

**Affiliations:** ^1^Department of Anesthesiology, Sun Yat-sen Memorial Hospital, Sun Yat-sen University, Guangzhou, China; ^2^Department of Gynecological Oncology, Sun Yat-sen Memorial Hospital, Sun Yat-sen University, Guangzhou, China; ^3^Guangdong Provincial Key Laboratory of Malignant Tumor Epigenetics and Gene Regulation, Sun Yat-sen Memorial Hospital, Sun Yat-sen University, Guangzhou, China; ^4^Medical Research Center, Sun Yat-sen Memorial Hospital, Sun Yat-sen University, Guangzhou, China

**Keywords:** postoperative neurocognitive disorder (PND), bioinformatics, competitive endogenous RNA network, function enrichment analysis, therapeutic target

## Abstract

Postoperative neurocognitive disorder (PND) is one of the most common postoperative neurological complications in aged patients, characterized by mental disorder, anxiety, personality changes, and impaired memory. At present, the molecular mechanism of PND remains largely unclear, and the ideal biomarker for clinical diagnosis and prognosis are lacking. Circular RNA (circRNA) and microRNA (miRNA), as unique non-coding RNAs, affecting the regulation of miRNAs on genes and further intervening in the progression of diseases through the sponge action between the two. Besides, it could be served as novel biomarkers in various diseases. In order to detect the differential expression profiles of genes caused by PND, a total of 26 18-month-old male C57BL/6 mice were randomly assigned to control group and PND group. Behavioral tests showed that mice in the PND group had impaired cognitive function compared with the control group. Three mice in each group were randomly selected to harvest the brain for analysis the expressions of circRNAs, miRNAs, and mRNAs in the prefrontal cortex by next-generation sequencing (NGS) technology. Differentially expressed genes, including 1192 circRNAs, 27 miRNAs, and 266 mRNAs were identified, and its accuracy was further confirmed by qRT-PCR. Bioinformatics analysis results suggested that neuroinflammation was the main pathological mechanism of PND. The construction of competitive endogenous RNA (ceRNA) networks and the identification of hub genes provided possible therapeutic targets for PND. Cinnarizine and Clemastine were predicted to have the potential therapeutic effects on PND. This is the first study to explore the differential expression profiles of genes and their regulation mechanisms in PND, our results provided new clues and targets for the treatment of this refractory disease.

## Introduction

Postoperative neurocognitive disorder (PND) is mainly manifested as reduced learning and memory ability, impaired social ability, and cognitive ability after non-brain operation (Evered et al., 2018). PND is a cognitive disorder disease that has attracted much attention in clinical work. Advanced age is considered as an independent risk factor of PND (Luo et al., 2019). Among aged patients over 60-year old, the overall incidence of PND is 10–15%, which is significantly higher than other postoperative complications such as pulmonary infection, stroke, and heart failure (Urits et al., 2019). PND increases the risk of Alzheimer’s disease and might lead to dementia (Evered et al., 2016), which seriously affects the quality of life of patients and imposes a heavy burden on society and patients’ families. Many studies have been performed to explore possible molecular mechanisms of PND. Neuroinflammation, neuronal apoptosis, amyloid-Aβ deposition, and hyperphosphorylation of tau proteins have been reported as main mediators in PND (Xie et al., 2013; Chen et al., 2019; Li M. et al., 2019). At present, the pathogenesis of PND remains elusive, and there is no effective intervention method.

The next-generation sequencing (NGS) technology, also known as high-throughput sequencing technology, can perform large-scale genomics researches. This application can reveal the potential pathological mechanism and provide new ideas for the treatment of disease (Jakel and Williams, 2020). Circular RNA (circRNA) and microRNA (miRNA) belong to non-coding RNAs. In terms of function, recent studies have shown that circRNA molecules are rich in miRNA binding sites and served as miRNA sponges, thereby releasing the inhibitory effect of miRNA on its target genes and increasing the expression level of target genes. This is the competitive endogenous RNA (ceRNA) mechanism. The ceRNA networks are widely involved in the process of various cognitive disorder diseases such as Alzheimer’s disease (Zhang et al., 2017; Wang et al., 2018b; Ma et al., 2019). Previous studies showed that different expressions of circRNAs were detected in the hippocampus of rats with PND (Cao et al., 2020). The differentially expressed circRNAs also had been determined in the serum of aged PND patients. Therefore, circRNAs may be related to the pathogenesis of PND (Gao et al., 2020). Meanwhile, the up-regulated expression of miRNA-572 in hippocampal neurons contributes to the recovery of cognitive function in rats with PND (Yu et al., 2015), and the miRNA-190a also plays a key role in the pathogenesis of PND (Liu et al., 2019). However, the differential expression profiles and possible ceRNA networks caused by PND remains unclear.

Therefore, in this study, the differential expression profiles in prefrontal cortex of aged mice with PND were analyzed by NGS. The ceRNA networks and hub genes were identified, and relevant functional enrichment analyses were performed by bioinformatics. In addition, some small molecular drugs with potential therapeutic effects on PND were predicted. These results might help provide new biomarkers and therapeutic targets for PND.

## Materials and Methods

### Animals

All the animal experiments in this study were approved by the Institutional Animal Care and Use Committee (Approval No.: SYSU-IACUC-2020-000326) and the Laboratory Animal Ethics Committee of Sun Yat-sen University.

The Sun Yat-sen University (Guangzhou, China) provided 18-month-old male C57BL/6 mice with a weight between 45 and 50 g for the experiment. The mice were randomly divided into two groups: isoflurane plus exploratory laparotomy (ISO + EL) group and control (CON) group. Each group had 13 mice. In a colony room with a temperature of 19–22°C, a humidity of 40–60%, and a light/dark cycle of 12 h (lighting time is 07:00–19:00), 5 mice were housed in each cage with access to food and water at will. The experiments began after all the animals had adapted to the environment for 2 weeks.

### Operation and Anesthesia

Isoflurane anesthesia plus exploratory laparotomy has been proved to be an effective method to construct PND model (Qiu et al., 2020). Before exploratory laparotomy, the mice were anesthetized by exposure to an oxygen chamber prefilled with 1.5% isoflurane for 30 min. A median incision approximately 2 cm in the abdomen was made to enter the abdominal cavity and explore the abdominal organs such as the liver, spleen, and intestine. Sterile 5-0 operation sutures were used to suture the peritoneum and skin. Operation was also performed under isoflurane anesthesia and lasted 30 min. At the end of operation and every day within 3 days after operation, 2.5% lidocaine cream was applied to the incision to alleviate the postoperative pain, and povidone iodine solution was applied to prevent infection. Mice in the control group were not treated with anesthesia or operation.

### Open Field

A black opaque plastic chamber (60 cm × 60 cm × 50 cm, ZH-ZFT, Anhui Zhenghua Biological Instrument equipment Co., Ltd., Anhui, China) was used as the open field arena. The open field test was performed 24 h after operation to evaluate the locomotor activity of the mice. Each mouse was placed in the center of the field and allowed to explore freely for 5 min while a video tracking system (Smart v3.0.06, Panlab Harvard Apparatus, Barcelona, ES) automatically recorded its movements and analyzed the total distance to assess the locomotor activity. Meanwhile, the time spent in the central area of mice were analyzed to evaluate whether postoperative anxiety occurred. During each test interval, the field was cleaned with 75% ethanol to eliminate feces and odors.

### Fear Conditioning

One day before the operation, each mouse was placed into the conditioning chamber (Freeze Monitor, San Diego Instruments, San Diego, CA, United States) and allowed to explore the room freely for 180 s. Then they were given a 30 s tone (70 db), followed by a 2 s foot shock (0.7 mA), and the next tone-shock stimulation cycle was entered at an interval of 60 s. A total of 3 cycles were performed. On postoperative days 1, 2 h after the open field test, each mouse was placed into the conditioning chamber without any tone or electrical stimulation for 360 s and the environment was identical with that before operation, the time of freezing behavior was recorded to evaluate the context-related memory. Two-hours later, they were placed into a new environment completely different from that before the operation and explored room freely for 180 s. Then they were given the same tone stimulation (without electrical stimulation) as before the operation, the time of freezing behavior was recorded to evaluate tone-related memory. Freezing behavior means there is no visible movement other than breathing. During each test interval, the conditioning chamber was cleaned with 75% ethanol to eliminate feces and odors.

### Barnes Maze

On postoperative days 2–6, we performed Barnes maze to evaluate the spatial learning and memory of mice. Each mouse was placed in the center of a circular platform (Anhui Zhenghua Biological Instrument equipment Co., Ltd., Anhui, China) with a diameter of 92 centimeters, which had 20 equally spaced holes. Among all the holes, only one was linked to a small dark recessed chamber. The mice were expected to find the hole and enter the dark chamber under the bright light (200 W). The mice were trained for 4 days with 3 trials per day, with each trial lasting 3 min, and an interval of 15 min between each trial. If the mice could not find the correct hole and enter the dark chamber within 3 min, they were guided to the correct location. During each test interval, the platform and dark chamber were cleaned with 75% ethanol to eliminate feces and odors. The last day, the escape latency was recorded and measured by a video tracking system (Smart v3.0.06, Panlab Harvard Apparatus, Barcelona, ES), which was used to evaluate the spatial learning and memory retention of mice.

### Harvesting of Tissue

Half an hour after the Barnes maze test, the mice were deeply anesthetized with isoflurane, and perfused transcardially with normal saline. We dissected the brain in the air to obtain the prefrontal cortex, and then frozen tissues in liquid nitrogen. Three cortical tissues were randomly selected from each group for subsequent sequencing analysis. The others were preserved for qRT-PCR.

### RNA Extraction and Purification for Next-Generation Sequencing

Total RNAs were extracted by miRNeasy Mini Kit (217004, Qiagen, GmBH, Germany) and checked for a RIN number to inspect RNA integrity by an Agilent Bioanalyzer 2100 (Agilent technologies, Santa Clara, CA, United States). Qualified total RNAs were further purified by RNAClean XP Kit (A63987, Beckman Coulter, Inc., Kraemer Boulevard Brea, CA, United States) and RNase-Free DNase Set (79254, Qiagen, GmBH, Germany). The purity of total RNAs were assessed by NanoDrop ND-2000 spectrophotometer (NanoDrop Technologies, Wilmington, DE, United States) and Agilent Bioanalyzer 2100 (Agilent Technologies, Santa Clara, CA, United States). The integrity of RNAs were determined by standard denaturing agarose gel electrophoresis.

### Library Preparation and Next-Generation Sequencing of circRNAs

Ribosomal RNAs (rRNAs) were removed from total RNAs, and then RNase R (RNR07250, Epicenter, United States) was used to digest linear RNAs. Subsequently, purified RNAs fragments were subjected to the first strand and second strand cDNA synthesis, and then adapter-ligated and enriched were performed by VAHTS Total RNA-seq (H/M/R) Library Prep Kit for Illumina (NR603-02, Vazyme, CHN). The quality and concentration of library were measured by the Qubit dsDNA HS Assay Kit (Q32854, Invitrogen, Carlsbad, CA, United States). Illumina HiSeq X ten platform was used for circRNAs sequencing to obtain the raw reads. Raw reads were filtered with Seqtk^1^ and obtained the clean reads. The results of clean reads were mapped to the reference genome by BWA-MEM^2^. CircRNAs were detected by CIRI (Beijing Institutes of Life Science, Beijing, China) (Gao et al., 2015), based on the circRNAs location information, the same circRNAs in each sample were merged, renumbered, and compared with the circBase database^3^. At present, the complete sequence cannot be obtained for most circRNAs (You et al., 2015), so we used SRPBM to normalize the clean reads (Li Y. et al., 2015). We used edgeR package of R software (version 3.6.0^4^) to analyze the different circRNAs between samples and get the *p*-value (Robinson et al., 2010). At the same time, we calculated the differentially expressed multiples based on the SRPBM value, which was Fold-change. The differential expressions of circRNAs were identified using the Limma package of R Software. The differentially expressed circRNAs were screened with | Fold-change| > 2 and *p*-value < 0.05 as the cut-off criteria. Volcano map and heatmap with differential expression of circRNAs were presented by ggpubr, ggthemes, and pheatmap packages of R software.

### Library Preparation and Next-Generation Sequencing of miRNAs

The adapter-ligated and enriched of purified total RNAs were performed by the NEBNext Multiplex Small RNA Library Prep Set for Illumina (Set 2) (E7580S, NEB, United States). After the amplification, the quality, and concentration of library were measured by the Qubit dsDNA HS Assay Kit (Q32854, Invitrogen, Carlsbad, CA, United States). Illumina HiSeq X ten platform was used for miRNAs sequencing to obtain the raw reads. Raw reads were filtered with fastx^5^ and obtained the clean reads. Clean reads of 18–40 nt length were compared with the reference genome by Bowtie (Langmead et al., 2009). And miRNAs were identified based on the location information of known miRNAs in miRBase (Kozomara and Griffiths-Jones, 2014). In order to make the expression levels of miRNAs between different miRNAs and different samples comparable, we normalized the number of clean reads mapped to each miRNA by the trimmed mean of *M* values (TMM) (Robinson and Oshlack, 2010), and then converted it into the transcripts per million (TPM, the formula is: the number of reads on a miRNA × 10^6^/total reads) for standardization of miRNAs expression (Mortazavi et al., 2008). We used edgeR package of R software to analyze the different miRNAs between samples and get the *p*-value. At the same time, we calculated the differentially expressed multiples based on the TPM value, which is Fold-change. The differential expressions of miRNAs were identified using the Limma package of R Software. The differentially expressed miRNAs were screened with | Fold-change| > 2 and *p*-value < 0.05 as the cut-off criteria. Volcano map and heatmap with differential expression of miRNAs were presented by ggpubr, ggthemes, and pheatmap packages of R software.

### Library Preparation and Next-Generation Sequencing of mRNAs

Similar to the circRNAs sequencing process, rRNAs were removed from total RNAs, purified RNAs fragments were subjected to the first strand and second strand cDNA synthesis, and then adapter-ligated and enriched were performed by VAHTS Total RNA-seq (H/M/R) Library Prep Kit for Illumina (NR603-02, Vazyme, CHN). The quality and concentration of library were measured by the Qubit dsDNA HS Assay Kit (Q32854, Invitrogen, Carlsbad, CA, United States). Illumina HiSeq X ten platform was used for mRNAs sequencing to obtain the raw reads. Raw reads were filtered with Seqtk and clean reads was obtained. Hisat2 spliced mapping algorithm was applied to perform genome mapping of pre-processed reads (Kim et al., 2015). In order to make data comparable, processed reads were transformed into fragments per kilobase of exon model per million mapped reads (FPKM, the formula is: total exon fragments/mapped reads/exon length) for the standardization of mRNAs expression. We used StringTie to count the number of fragments of each gene after Hisat2 comparison (Pertea et al., 2015, 2016), then used TMM value to make normalize, and finally used perl (version 5.32.1^6^) to calculate the number of fragments for each gene FPKM value. EdgeR package of R software was also used to analyze the different mRNAs between samples, and the differentially expressed multiples were calculated based on the FPKM value. The differential expressions of mRNAs were identified using the Limma package of R Software. The differentially expressed mRNAs were screened with | Fold-change| > 2 and *p*-value < 0.05 as the cut-off criteria. Volcano map and heatmap with differential expression of mRNAs were presented by ggpubr, ggthemes and pheatmap packages of R software.

### Functional Enrichment Analysis

Gene ontology (GO) enrichment analysis is a commonly used method of gene annotation, including biological pathways (BP), molecular functions (MF), and cell components (CC). Kyoto Encyclopedia of Genes and Genomes (KEGG) pathway enrichment analysis was used to identify the key biological pathways involved in candidate genes. The clusterProfiler package of R software was used to perform GO and KEGG pathway enrichment analysis was based on the differentially expressed mRNAs with adjusted *p*-value < 0.05 as the cut-off criterion. The *p*-value was adjusted by false discovery rate (FDR) method.

### Identification of Protein-Protein Interaction Networks and Hub Genes

The Search Tool for the Retrieval of Interacting Genes (STRING, version 11.0^7^) database was used to predict protein-protein interactions (PPI) between candidate genes. In order to explore the potential correlation between these differentially expressed mRNAs, we used STRING to obtain the data of PPI. The protein-protein minimum required interaction score was set to 0.7, indicating high confidence. Furthermore, hub genes were identified based on the count of interaction pairs between proteins. Cytoscape (version 3.8.0, San Diego, CA, United States) was used to visualize the PPI network data and identify the hub genes.

### Construction of ceRNA Networks

The multiMiR package of R software collects nearly 50 million human and mouse miRNA-mRNA interaction records from 14 different databases (Ru et al., 2014). We used multiMiR to retrieve miRNA-mRNA interactions. Based on 108 CLIP-Seq (PAR-CLIP, HITS-CLIP, iCLIP, and CLASH) data sets, starBase database (v2.0^8^) identified approximately 10,000 ceRNA pairs from 37 independent studies. The starBase database was applied to retrieve miRNA-circRNA interactions (Li J. H. et al., 2014). The obtained genes were intersected with the sequencing results via an online Venn tool^9^. Based on the inter-relationships of circRNAs-miRNAs-mRNAs, ceRNA networks were then constructed by Cytoscape to visualize the genes regulation networks.

### cDNA Synthesis and Quantitative Real-Time PCR (qRT-PCR)

To verify the accuracy and reliability of the sequencing results, 5 circRNAs (circRalgapa1, cird3, circPpme1, circKansl1l, and circRnf150), 2 miRNAs (miR-200b-3p and miR-141-3p), and 5 mRNAs (Slco1b2, Ino80d, Chrnb1, Sncg, and Agt) were randomly selected for qRT-PCR, which were selected from the ceRNA network, the most obvious differential expression genes and the hub genes. Total RNA was extracted from the prefrontal cortex of the PND group and CON group (*n* = 10, each group) using RNA Quick Purification kit (RN001, Esscience, CHN). For circRNAs and mRNAs, the reverse transcription to cDNA was achieved with Hifair^®^ III 1st Strand cDNA Synthesis SuperMix for qPCR (11141ES60, Yeasen, CHN). For miRNAs, the reverse transcription was achieved with Bulge-Loop miRNA qRT-PCR Starter Kit (C10211-1, RiboBio, and CHN). And qRT-PCR was conducted with Roche LightCycler 480 II Real-Time PCR System (Roche, United States). The relative expression levels were calculated by the 2^–ΔΔCT^ method. GAPDH was used as an internal reference gene for circRNAs and mRNAs expression, and U6 was used as an internal reference gene for miRNAs expression. Sequence-specific primers for U6 and miRNAs were synthesized by RiboBio (Guangzhou, China). The sequences of primers of mRNAs, circRNAs, and GAPDH were presented in Table 1.

**TABLE 1:** The primer sequences used for qRT-PCR.

Gene	Primer sequences (5′–3′)
circRalgapa1	Forward, GATGACTCTCTGACCAAAAAATGA
	Reverse, TGTTAGCATAGCAGGACCACC
circPsd3	Forward, GGATTGCACGATTCTGCCAA
	Reverse, AAGTGCTTCCTCCATGATCACCT
circPpme1	Forward, GGAGAATGAAACTGGCAAGGATAC
	Reverse, TTCCGTCCAGGGCGTGAA
circKansl1l	Forward, GGACTCTGACGCCACCGATA
	Reverse, CTTAAACTCTTAAAAACATACTGCCA
circRnf150	Forward, CAGGAAGGGTGACAAGGAAAC
	Reverse, TGACCAGTAGCTATCGTGTACGG
Slco1b2	Forward, GTTGTGTCAGCGGCTCTGGT
	Reverse, GCATTTGTCTCTTGGGCATTC
Ino80d	Forward, CTCCGCTGCATAAACTACCCT
	Reverse, GGACTGCTGCTCAACCAAGAA
Chrnb1	Forward, CTCCAACTATGATAGCTCGGTGA
	Reverse, CAGGTCTAAGTACACCTTTGTGC
Sncg	Forward, AAAGACCAAGCAGGGAGTAACG
	Reverse, GACCACGATGTTTTCAGCCTC
Agt	Forward, TCTCCTTTACCACAACAAGAGCA
	Reverse, CTTCTCATTCACAGGGGAGGT
GAPDH	Forward, GGGTCCCAGCTTAGGTTCAT
	Reverse, TACGGCCAAATCCGTTCACA

### Prediction of Potential Therapeutic Small Molecular Drugs

Connectivity Map (CMap^10^) was used to measure connectivity between disease gene expression characteristics and compound induced gene expression profiles, which has become a valuable tool in understanding drug mechanism of action and discovering new indications for drugs (Cheng et al., 2014). These mRNAs significantly up-regulated or down-regulated were uploaded onto the CMap database to predict the small molecular drugs with potential therapeutic effects on PND.

### Statistical Analysis

All results were expressed as mean ± SD. Statistical analyses were performed using the GraphPad Prism 8.0 (San Diego, CA, United States) and R software. Student’s *t*-test was used to determine the statistical significance between two groups. A *p*-value < 0.05 was considered statistically significant.

## Results

### Isoflurane Plus Exploratory Laparotomy Caused PND in Aged Mice

**FIGURE 1:**
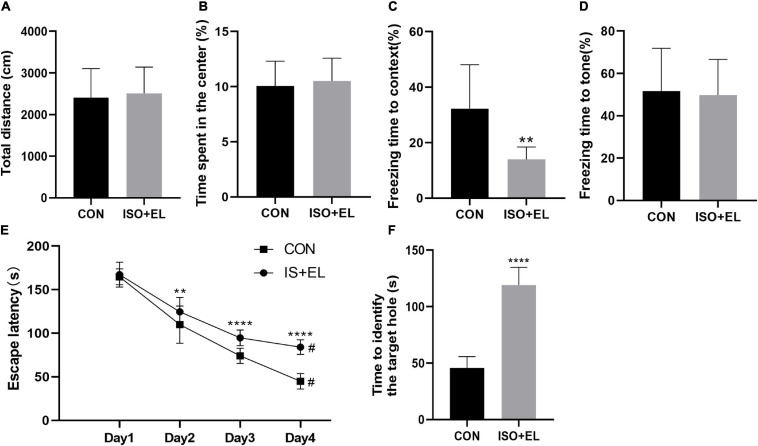
Anesthesia and operation-induced PND in aged mice. **(A)** Total distance of aged mice in open field test. **(B)** Time spent in the central area of aged mice in open field test. **(C)** Performance of aged mice in context test. **(D)** Performance of aged mice in tone test. **(E)** Performance of aged mice in Barnes maze training phase. **(F)** Performance of aged mice in Barnes maze test phase. The data are presented as mean ± S.D (*n* = 10). **P < 0.01 compared with the CON group; ****P < 0.001 compared with the CON group, ^#^*P* < 0.05 compared with the corresponding data in the first trial on day 1.

Our previous study has confirmed that isoflurane plus exploratory laparotomy (ISO + EL) successfully induced PND model in aged mice (Wang et al., 2018a). In the present study, there was no statistical difference in the total distance between the control (CON) group and the ISO + EL group at the same time window in the open field test. The result indicating that anesthesia and operation factors have no effect on the locomotor activity of the aged mice (Figure 1A). At the same time, the time spent in the central area had no statistically different, indicating that anesthesia and operation factors did not induce postoperative anxiety behaviors of aged mice, which was consistent with the results of previous studies (Qiu et al., 2020; Figure 1B). Fear conditioning test was divided into two parts: the context-related test and the tone-related test. In the context test, we found that aged mice in the ISO + EL group performed less freezing behavior in the new context than mice in the CON group, which indicated anesthesia and operation factors impaired the context-related memory of aged mice (Figure 1C). However, in the tone test, we found that the performance of mice was similar between in the ISO + EL group and in the CON group (Figure 1D). During the training phase of the Barnes maze, compared with day one, the time required for all mice to find the target hole significantly decreased on day four (Figure 1E). This suggested that all the mice got performance development during the training process. However, we found that mice in the ISO + EL group took longer time to identify the target hole than mice in the CON group during test phase (Figure 1F). This suggested that anesthesia and operation factors impaired the spatial learning and memory in older mice. In general, ISO + EL impaired the spatial learning and memory and the context-related memory in aged mice, which met the criteria of PND, our results were similar to previous studies (Lin and Zuo, 2011; Li J. et al., 2019; Lin et al., 2020). Therefore, the ISO + EL group was PND group.

### Identification of Differentially Expressed circRNAs

**FIGURE 2:**
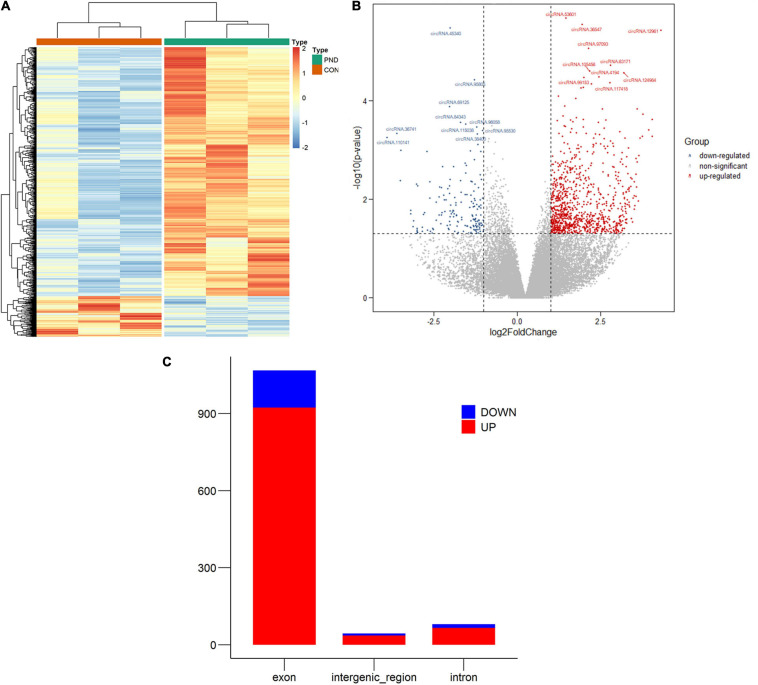
Altered expression profile of circRNAs in the prefrontal cortex of aged mice. **(A)** Hierarchical clustering analysis of all differentially expressed circRNAs in the prefrontal cortex of CON group and PND group: the red represents a higher fold change, while the blue represents a lower fold change. **(B)** The volcano plots of all the detected circRNAs in both the CON and PND groups: The vertical line represents to | log_2_ (fold change) | > 1 up and down, respectively, and the horizontal line corresponds to a *p*-value of 0.05 (–log_10_ scaled). The red points represent the significantly up-regulated circRNAs and the blue points represent the significantly down-regulated circRNAs. The top ten differentially expressed circRNAs were also indicated in the figure. **(C)** The gene sources of differentially levels of circRNAs. The red represents the significantly up-regulated circRNAs and the blue represents the significantly down-regulated circRNAs.

In this study, NGS technology was used to identify the differentially expressed circRNAs in the prefrontal cortex of aged mice with PND. A total of 71481 circRNAs were identified. Compared with the CON group, we had screened 1192 differentially expressed circRNAs in the PND group, which included 1025 up-regulated circRNAs and 167 down-regulated circRNAs. The hierarchical clustering heatmaps revealed distinguishable circRNAs expression profiles (Figure 2A), and the volcano plots showed a significant difference in the expression levels of circRNAs between the PND group and CON group (Figure 2B). In the prefrontal cortex of aged mice with PND, most of the differentially expressed circRNAs were originated from exons (Figure 2C).

### Identification of Differentially Expressed miRNAs

**FIGURE 3:**
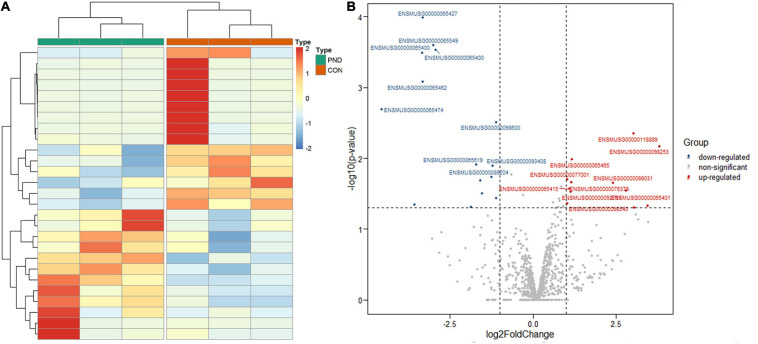
Altered expression profile of miRNAs in the prefrontal cortex of aged mice. **(A)** Hierarchical clustering analysis of all differentially expressed miRNAs in the prefrontal cortex of CON group and PND group: the red represents a higher fold change, while the blue represents a lower fold change. **(B)** The volcano plots of all the detected miRNAs in both the CON and PND groups: The vertical line represents to | log_2_ (fold change) | > 1 up and down, respectively, and the horizontal line corresponds to a *p*-value of 0.05 (–log_10_ scaled). The red points represent the significantly up-regulated miRNAs and the blue points represent the significantly down-regulated miRNAs. The top ten differentially expressed miRNAs were also indicated in this figure.

Compared with the CON group, a total of 1189 miRNAs were identified in the prefrontal cortex of aged mice with PND. We had screened 27 differentially expressed miRNAs in the PND group, which included 12 up-regulated miRNAs and 15 down-regulated miRNAs. The hierarchical clustering heatmaps revealed distinguishable miRNAs expression profiles (Figure 3A), and the volcano plots showed a significant difference in the expression levels of miRNAs between the PND group and CON group (Figure 3B).

### Identification of Differentially Expressed mRNAs

**FIGURE 4:**
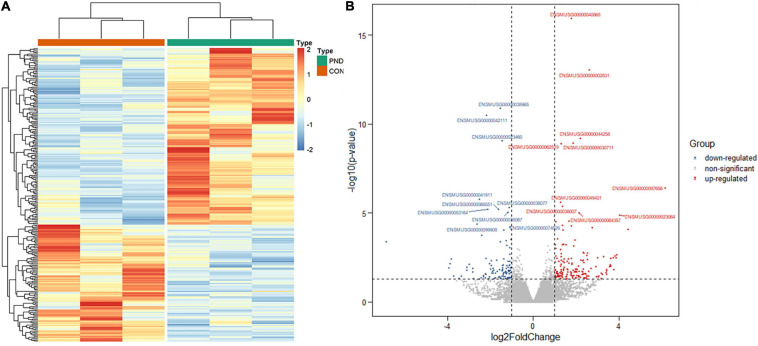
Altered expression profile of mRNAs in the prefrontal cortex of aged mice. **(A)** Hierarchical clustering analysis of all differentially expressed mRNAs in the prefrontal cortex of CON group and PND group: the red represents a higher fold change, while the blue represents a lower fold change. **(B)** The volcano plots of all the detected mRNAs in both the CON and PND groups: The vertical line represents to | log_2_ (fold change) | > 1 up and down, respectively, and the horizontal line corresponds to a *p*-value of 0.05 (–log_10_ scaled). The red points represent the significantly up-regulated mRNAs and the blue points represent the significantly down-regulated mRNAs. The top ten differentially expressed mRNAs were also indicated in this figure.

Compared with the CON group, a total of 21006 mRNAs were identified in the prefrontal cortex of aged mice with PND. We had screened 266 differentially expressed mRNAs in the PND group, which included 160 up-regulated mRNAs and 106 down-regulated mRNAs. The hierarchical clustering heatmaps revealed distinguishable mRNAs expression profiles (Figure 4A), and the volcano plots showed mRNAs levels with significant difference between the PND group and CON group (Figure 4B).

### Bioinformatics Analysis of Differentially Expressed mRNAs

**FIGURE 5:**
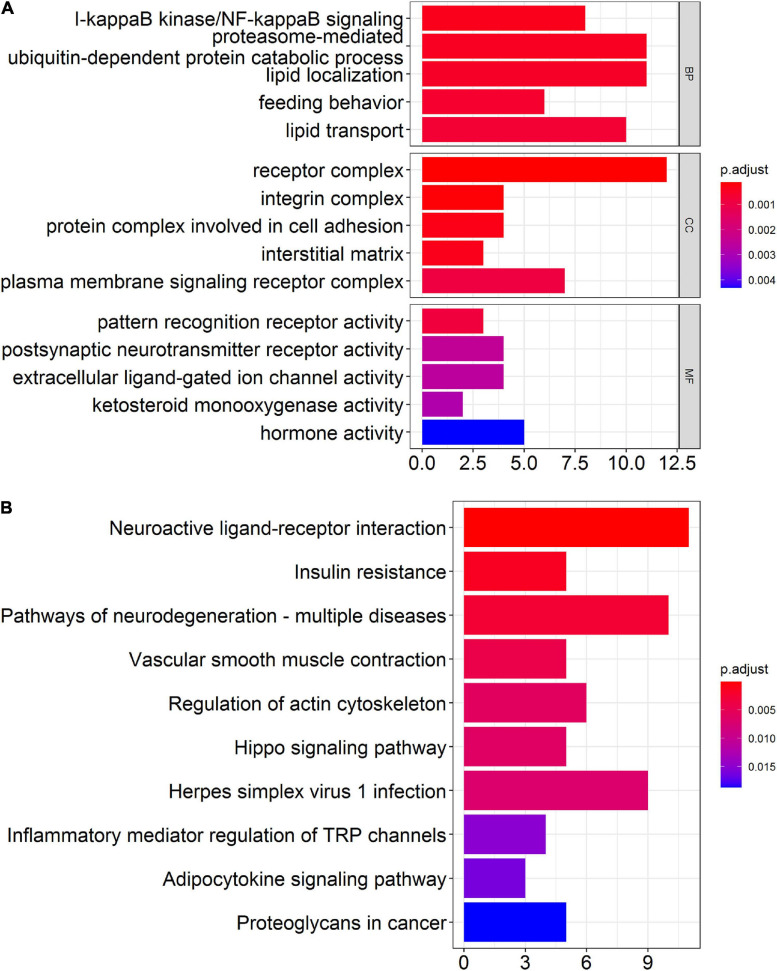
Bioinformatics analysis of differentially expressed mRNAs in the prefrontal cortex of aged mice. **(A)** Gene ontology (GO) analysis of the differentially expressed mRNAs in the prefrontal cortex of aged mice. **(B)** Kyoto Encyclopedia of Genes and Genomes (KEGG) pathway analysis of the differentially expressed mRNAs in the prefrontal cortex of aged mice.

Although differentially expressed circRNAs and miRNAs have been identified in prefrontal cortex, proteins transcribed from mRNAs actually play key biological functions *in vivo* and *in vitro*. Therefore, we performed GO enrichment analysis and KEGG pathway analysis to predict the possible biological functions of the differentially expressed mRNAs. The top GO terms in the biological processes (BP) were included as below: I-kappaB kinase/NF-kappaB signaling, proteasome-mediated ubiquitin-dependent protein catabolic process, lipid localization, feeding behavior, and lipid transport. The top GO terms in the cellular components (CC) were listed as below: receptor complex, integrin complex, and protein complex involved in cell adhesion, interstitial matrix, and plasma membrane signaling receptor complex. The top GO terms in the molecular function (MF) were include as below: pattern recognition receptor activity, postsynaptic neurotransmitter receptor activity, extracellular ligand-gated ion channel activity, ketosteroid monooxygenase activity, and hormone activity (Figure 5A). Through KEGG pathway analysis, the top 10 enriched pathways found as below: neuroactive ligand-receptor interaction, insulin resistance, pathways of neurodegeneration-multiple diseases, vascular smooth muscle contraction, regulation of actin cytoskeleton, hippo signaling pathway, herpes simplex virus 1 infection, inflammatory mediator regulation of TRP channels, adipocytokine signaling pathway, and proteoglycans in cancer (Figure 5B).

### Construction of PPI Network

**FIGURE 6:**
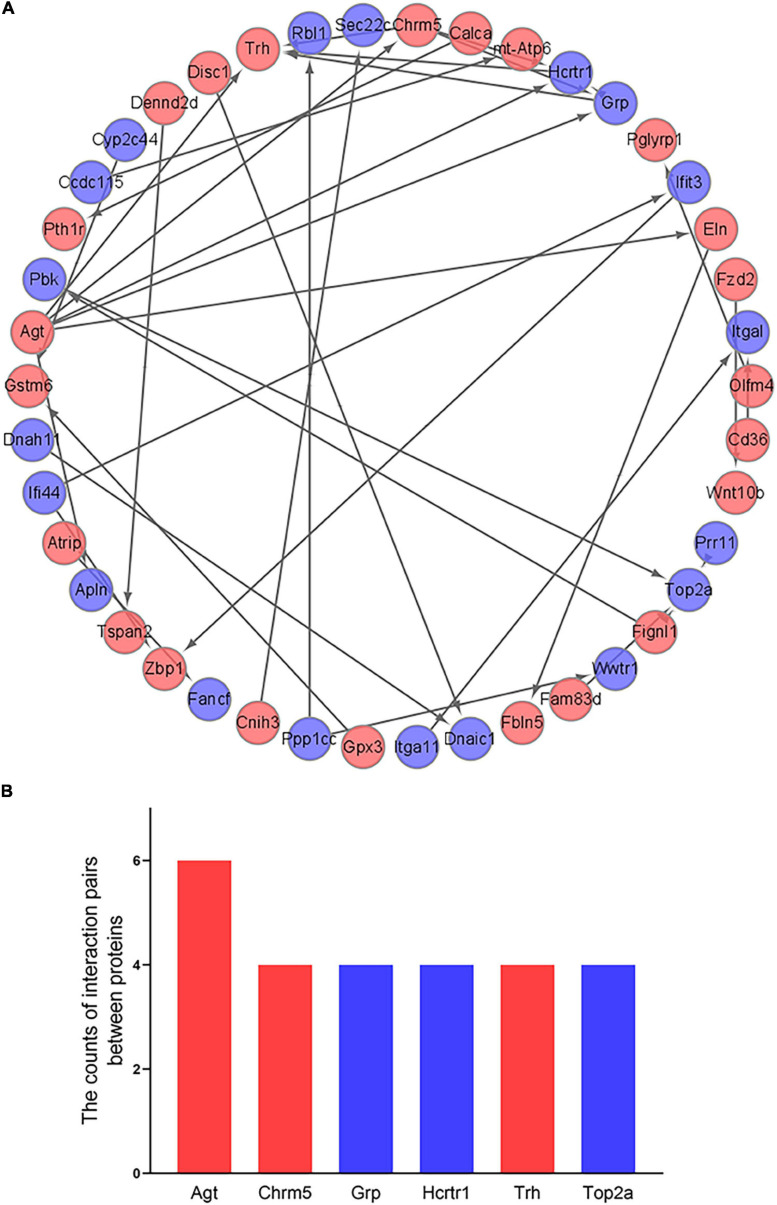
Construction of PPI network and identification of hub genes. **(A)** Visualization of PPI network. The red points represent 23 up-regulated genes and the blue points represent 19 down-regulated genes. **(B)** Identification of the top 6 hub genes. The red bars represent up-regulated genes and the blue bars represent down-regulated genes.

The 266 differentially expressed mRNAs were upload onto the STRING database to explore the inter-relationships among the different proteins, a total of 198 nodes and 36 edges were obtained. We visualized these data by Cytoscape. And a total of 42 mRNAs were mapped into the network, which included 23 up-regulated mRNAs and 19 down-regulated mRNAs (Figure 6A). And then, we identified the top 6 hub genes (Agt, Chrm5, Grp, Hcrtr1, Trh, and Top2a) by the count of interaction pairs between different proteins, indicating that these genes may have important roles in PND (Figure 6B).

### Construction of ceRNA Networks

**FIGURE 7:**
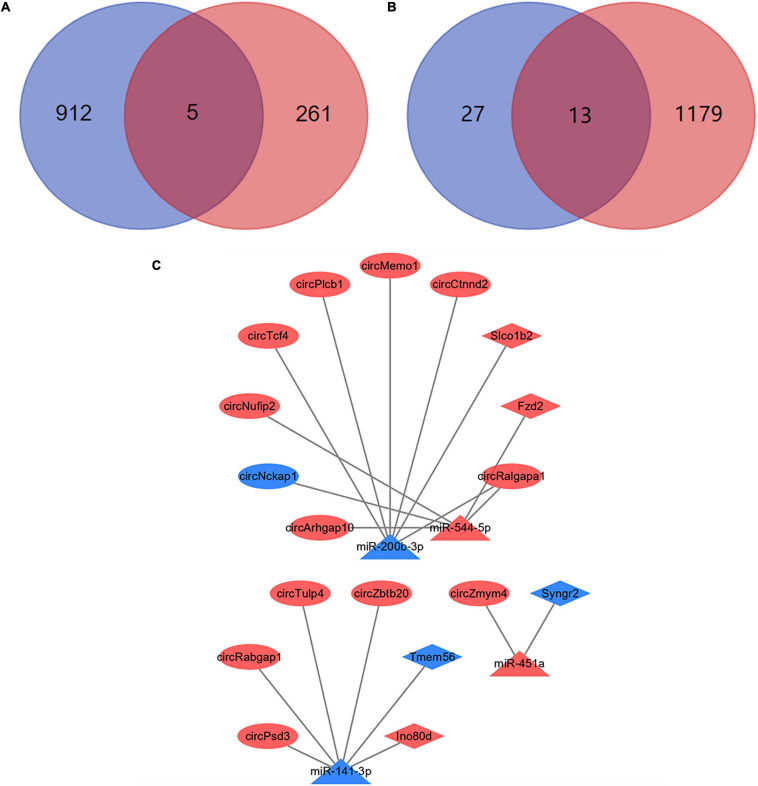
Construction of ceRNA networks. **(A)** A total of 5 mRNAs were identified with miRNA targeting binding sites. The blue circle represents the results of bioinformatics databases and the red circle represents the NGS results. **(B)** A total of 13 circRNAs were identified with miRNA targeting binding sites. The blue circle represents the results of bioinformatics databases and the red circle represents the NGS results. **(C)** Visualization of ceRNA networks. The red marks represent the up-regulated genes and the blue marks represent the down-regulated genes.

Most circRNAs can act as miRNAs sponges to eliminate the inhibition of miRNA on their downstream mRNAs expressions. And this is the main mechanism of ceRNA networks. Based on differentially expressed miRNAs, 917 downstream mRNAs were obtained by the multiMiR package of R software. We intersected these mRNAs with the differentially expressed mRNAs which obtained by NGS. And 5 mRNAs were identified as the downstream mRNAs of miRNAs (Figure 7A). Their corresponding relationships with miRNAs were shown in Table 2. By the starBase database, 40 circRNAs which were obtained could competitively bind with the differentially expressed miRNAs. Meanwhile, 13 circRNAs were identified as the ceRNAs of miRNAs (Figure 7B). The possible corresponding relationships between miRNAs and circRNAs were shown in Table 3. Based on the relationships between miRNAs-mRNAs and miRNAs-circRNAs obtained by databases, combined with the expression trend of genes, a ceRNA network were visualized by Cytoscape (Figure 7C). In this network, we found that the circTcf4, circPlcb1, circMemo1, circCtnnd2, circRalgapa1/miR-200b-3p/Slco1b2 axes, and the circPsd3, circRabgap1, circTulp4, circZbtb20/miR-141-3p/Ino80d axes conform to the general gene expression law of ceRNA network, which might be involved in the mechanisms of PND regulation.

**TABLE 2:** Correspondence relationships between miRNAs and mRNAs.

miRNA	mRNA	Entrez ID	ENSEMBL
mmu-miR-451a	Syngr2	20973	ENSMUSG00000048277
mmu-miR-200b-3p	Slco1b2	28253	ENSMUSG00000030236
mmu-miR-544-5p	Fzd2	57265	ENSMUSG00000050288
mmu-miR-141-3p	Tmem56	99887	ENSMUSG00000028132
mmu-miR-141-3p	Ino80d	227195	ENSMUSG00000040865

**TABLE 3:** Correspondence relationships between miRNAs and circRNAs.

miRNA	circRNA	Position	Type
mmu-miR-451a	circZmym4	chr4:126925560-126925584	Exon
mmu-miR-200b-3p	circPlcb1	chr2:135370541-135370564	Exon
mmu-miR-200b-3p	circRalgapa1	chr12:55676680-55676706	Exon
mmu-miR-200b-3p	circRalgapa1	chr12:55665649-55665670	Exon
mmu-miR-200b-3p	circCtnnd2	chr15:30683345-30683368	Exon
mmu-miR-200b-3p	circMemo1	chr17:74241942-74241961	Exon
mmu-miR-200b-3p	circTcf4	chr18:69564099-69564119	Exon
mmu-miR-544-5p	circNckap1	chr2:80520518-80520537	Exon
mmu-miR-544-5p	circArhgap10	chr8:77358567-77358587	Exon
mmu-miR-544-5p	circNufip2	chr11:77691823-77691843	Exon
mmu-miR-544-5p	circRalgapa1	chr12:55677150-55677171	Exon
mmu-miR-141-3p	circRabgap1	chr2:37540423-37540444	Exon
mmu-miR-141-3p	circPsd3	chr8:67964163-67964184	Exon
mmu-miR-141-3p	circZbtb20	chr16:43577118-43577140	Intron
mmu-miR-141-3p	circTulp4	chr17:6137458-6137479	Exon

### Validation of the Differentially Expressed Genes

**FIGURE 8:**
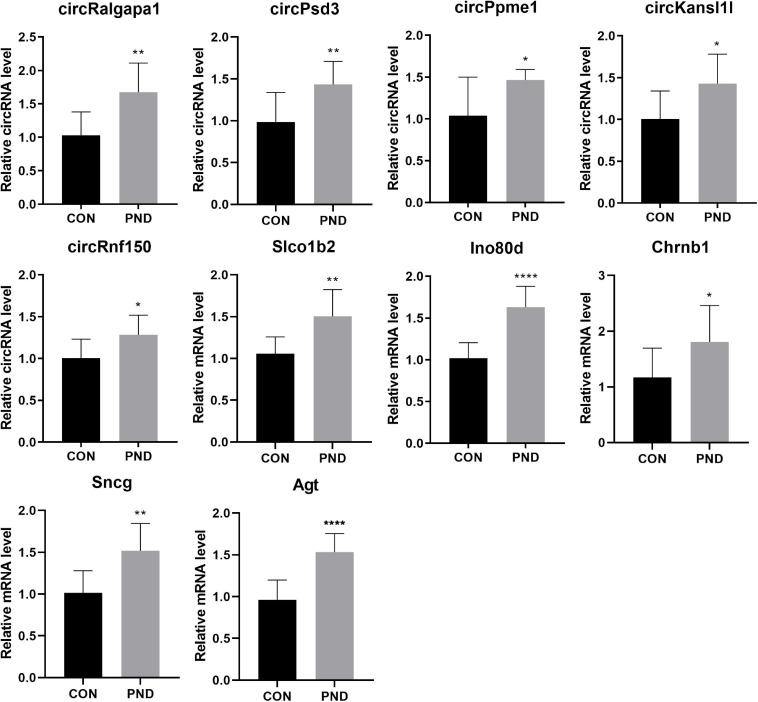
The validation results of circRalgapa1, circPsd3, circPpme1, circKansl1l, circRnf150, Slco1b2, Ino80d, Chrnb1, Sncg, and Agt by qRT-PCR in CON and PND groups (*n* = 10, each group). *P < 0.05 compared with the CON group, **P < 0.01 compared with the CON group, ****P < 0.005 compared with the CON group.

**FIGURE 9:**
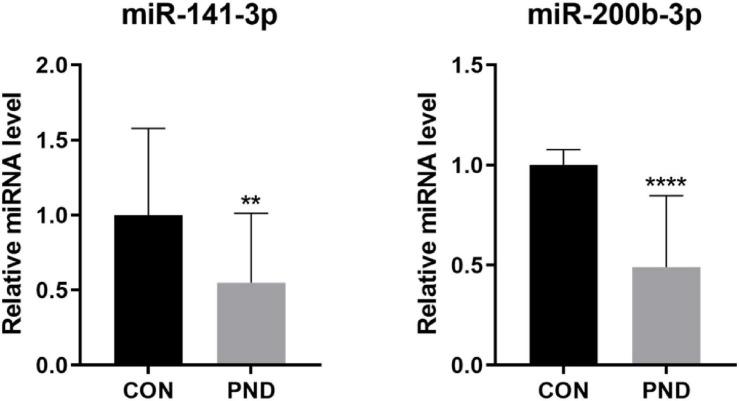
The validation results of miR-200b-3p and miR-141-3p by qRT-PCR in CON and PND groups (*n* = 10, each group). ****P < 0.001 and **P < 0.01 compared with the CON group.

To validate the accuracy and reliability of the NGS results, we randomly selected 5 circRNAs, 2 miRNAs, and 5 mRNAs for qRT-PCR. Compared with CON group, the expressions of circRalgapa1, circPsd3, circPpme1, circKansl1l, cinrcRnf150, Slco1b2, Ino80d, chrnb1, Sncg, and Agt in PND group were significantly up-regulated (Figure 8). The expressions of miR-200b-3p and miR-141-3p were significantly down-regulated (Figure 9). The results of qRT-PCR largely verified the accuracy of NGS results and provided potential therapeutic targets for the treatment of PND.

### Prediction of the Potential Therapeutic Drugs

**FIGURE 10:**
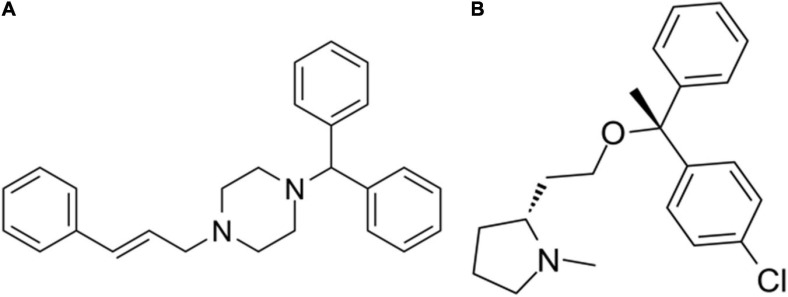
The chemical structure of potential therapeutic drugs. **(A)** The chemical structure of Cinnarizine. **(B)** The chemical structure of Clemastine.

The differentially expressed mRNAs in the prefrontal cortex of aged mice with PND were upload onto the CMap database to predict the small molecular drugs that have potential therapeutic effects on PND. Negative drug’s mean value indicating the drug has a potential to improve the cognitive function of PND and reverse the expression of differentially expressed genes included in the ceRNA network. The top ten small molecular drugs were shown in Table 4. We found that Cinnarizine and Clemastine had the highest negative mean value. These results indicated that these two compounds might have potential therapeutic effects on PND, and the chemical structure of these two drugs were shown in Figures 10A,B.

**TABLE 4:** The top 10 small molecular drugs with high negative correlations with PND.

Rank	CMap name	Mean	N	Enrichment	*P*-value	Specificity	Percent non-null
1	Cinnarizine	−0.591	4	−0.866	0.00062	<0.01	100
2	Sulfamonomethoxine	−0.39	4	−0.848	0.00099	<0.01	75
3	Streptomycin	−0.491	4	−0.841	0.00117	<0.01	75
4	Clemastine	−0.673	3	−0.909	0.00132	<0.01	100
5	Tranexamic Acid	−0.417	5	−0.749	0.00186	0.0336	60
6	Podophyllotoxin	−0.297	4	−0.804	0.00282	0.0588	50
7	Ikarugamycin	−0.41	3	−0.847	0.00707	0.0227	66
8	Vigabatrin	−0.349	3	−0.845	0.00743	0.0141	66
9	Mafenide	−0.462	5	−0.673	0.00869	0.012	80
10	Nilutamide	−0.543	4	−0.715	0.01339	0.0107	75

## Discussion

Among all the risk factors closely associated with PND, age is the only one factor associated with long-term PND (more than 3 months after operation) (Le et al., 2014). Meanwhile, advanced age is considered as an independent risk factor of PND (Luo et al., 2019), which is not conducive to neurogenesis, neuron survival, and restoration of neuronal function following injury (Koks et al., 2016). Based on these theory, 18-month-old male C57BL/6 mice were used as experimental subjects in this study. The PND animal model was established by exploratory laparotomy plus isoflurane anesthesia, which has been generally proved to be effective in previous studies (Wang et al., 2018a; Qiu et al., 2020). The hippocampus and the cortex, especially the prefrontal cortex, play important roles in regulating cognitive function (Watanabe and Funahashi, 2014; Mansouri et al., 2017; Martorell et al., 2019). In behavioral tests, we found that the spatial learning memory and context-related memory were impaired in aged mice after anesthesia and operation without any post-operative anxious behavior. The phenomenon is regulated by both the hippocampus and the prefrontal cortex (Raybuck and Lattal, 2011), which strongly supports our view that anesthetic and operating factors contribute to the development of PND in aged mice.

As a serious perioperative neurological complication, PND impair patients’ postoperative memory, attention, language comprehension, and social skills, increasing the incidence of dementia and affecting the prognosis of patients (Evered et al., 2016, 2018). Although PND has a profound impact on patients, it is difficult to determine the specific pathogenesis. Many pathological processes have been involved in PND, such as, neuroinflammation (Luo et al., 2019), mitochondrial dysfunction, oxidative stress (Netto et al., 2018), the blood-brain barrier damage (Zhu et al., 2018), neurotrophic support impairment (Fan et al., 2016), and synaptic damage (Xiao et al., 2018). Due to its complex pathogenesis, there is a lack of effective treatments. The application of NGS technology has greatly improved our cognition to identify and diagnose the causes of disease (Rego and Snyder, 2019). Therefore, the NGS technology help us to obtain new clues and treatment targets for PND.

Currently, by RNA sequencing in the hippocampus of aged rats with PND, some studies have identified the differentially expressed circRNAs. And these circRNAs are related to the P53 signaling pathway and the NF-KB pathway (Cao et al., 2020). Like PND, Alzheimer’s disease is also associated with progressive impairment of cognitive function. Differential expression of circRNAs has been found in the hippocampus of Alzheimer’s disease patients and mouse models (Lu et al., 2019; Lo et al., 2020; Ma et al., 2020). These results indicated that the differentially expressed circRNAs were related to cognitive function changes. Moreover, the role of miRNAs in PND has been analyzed in many studies. The miR-572 improves early postoperative cognitive dysfunction by down-regulating NCAM1 (Yu et al., 2015). Meanwhile, the miR-190a improves postoperative cognitive dysfunction in mice by regulating the expression of Tiam1 (Liu et al., 2019). The miRNA-146a protects mice from cognitive impairment caused by operation by inhibiting the neuroinflammation (Chen L. et al., 2019). These results indicate that genes including circRNAs and miRNAs are closely related to PND.

Many circRNAs contain miRNAs binding sites and have the characteristics of miRNA sponge. The inhibition of miRNAs on downstream gene expression is eliminated by binding to target miRNAs. This is the ceRNA mechanism (Thomson and Dinger, 2016), which plays a key role in gene transcription. In this study, 1192 circRNAs and 27 miRNAs with differential expression were identified. Based on our sequencing results, 14 ceRNA pairs were obtained by bioinformatics databases. In addition, the results suggested that not all circRNAs and miRNAs had ceRNA relationships, and the function of most circRNAs need to be further explored. The miRNAs are short non-coding RNAs that regulate gene expression through mRNA degradation or translation inhibition (Zhang et al., 2020). Based on 266 differentially expressed mRNAs, 5 miRNA-mRNA axes were further identified and circRNA-miRNA-mRNA networks were constructed. Genes in these networks, together with other differentially expressed genes, may play key roles in the regulation of the pathogenesis of PND in aged mice.

From our sequencing results, it is not difficult to observe that among all differentially expressed genes, only a small part of genes can form a ceRNA network. In other words, the upstream of differentially expressed mRNAs, such as miRNAs and circRNAs, may not be differentially expressed. This also reflected that the regulation of genes expression in the brain is an extremely complex process.

Gene ontology enrichment and KEGG pathway analysis were performed to explore the roles of differentially expressed mRNAs in the pathogenesis of PND. In the terms of BP, we found that I-kappaB kinase/NF-kappaB signaling was significantly enriched. NF-kappaB signaling is a classical well-known neuroinflammatory signaling pathway, which induces the occurrence and development of PND (Zhu et al., 2017; Cao et al., 2018). The over-activation of NF-kappaB signaling leads to blood-brain barrier damage and the brain injury aggravation (Li Z. et al., 2016). The inhibition of NF-kappaB signaling reduces the neuroinflammation and oxidative stress, which could improve the cognitive impairment associated with PND (Yang and Yuan, 2018). In the terms of MF, we found that postsynaptic neurotransmitter receptor activity was significantly enriched. Postsynaptic neurotransmitter receptor transport is the basic mechanism for the dynamic regulation of synaptic strength, which is crucial for the formation and regulation of neuronal synaptic function (Papadopoulos et al., 2017). The dysregulation of the formation and regulation of neuronal synapse function is closely related to the impaired cognitive function of PND patients (Gao et al., 2021). In the results of KEGG pathway analysis, insulin resistance, pathways of neurodegeneration-multiple diseases, hippo signaling pathway and inflammatory mediator regulation of TRP channels were significantly enriched. Insulin resistance accelerated the development of PND (Tang et al., 2017; He et al., 2019), intranasal insulin treatment prevents anesthesia-induced cognitive impairments (Li X. et al., 2019). Neurodegeneration is one of the main features of PND (Pan et al., 2016). Hippo signaling pathway is involved in neuroinflammation, neuronal cell differentiation, and neuronal death (Cheng et al., 2020), and its intervention might contribute to the treatment of cognitive dysfunction (Yu et al., 2019). TRP channels are expressed in various cell types in the brain and playing a pathological role in a variety of neuroinflammatory diseases (Hu et al., 2020). The inhibitor of TRP channels significantly improve cognitive dysfunction in different diseases (Hazalin et al., 2020; Hu et al., 2020; Thapak et al., 2020). These results reveal the mechanism by which differentially expressed genes in the prefrontal cortex may regulate PND.

Based on the differentially expressed mRNAs, we constructed a PPI network to identify the hub genes of PND. The results indicated that Agt had the largest count of interaction pairs between proteins. The full name of Agt is Angiotensinogen, which is a basic component of the renin-angiotensin system. Its overexpression reflected the activation of renin-angiotensin system, which is closely related to the induction of PND (Li Z. et al., 2014). The expression of Agt was significantly up-regulated in the cerebrospinal fluid of patients with Alzheimer’s disease, which was associated with damage to the blood-brain barrier and impairment of cognitive function (Mateos et al., 2011). The activation of renin-angiotensin system can lead to cognitive decline and brain damage caused by chronic cerebral ischemia (Dong et al., 2011). The identification of hub genes provided potential therapeutic targets for PND.

Meanwhile, Cinnarizine and Clemastine were predicted to have potential therapeutic effects on PND. Cinnarizine is an antihistamine and T-type calcium channel inhibitor that promotes cerebral blood flow. Clomastine is a histamine H1 receptor antagonist. The application of antihistamines could ameliorated cognitive function in mice with Alzheimer’s disease (Steele et al., 2009; Wang et al., 2011). There are many similarities between PND and Alzheimer’s disease in view of pathogenesis, such as oxidative stress, neurodegeneration, and neuroinflammation. It has been reported that Cinnarizine can improve haloperidol-induced memory impairment and oxidative stress (Abdel-Salam et al., 2012). Clemastine shows more potential in cognitive enhancement than Cinnarizine. It could reduce the IL-1β mediated neuroinflammation (Liu et al., 2016; Xie et al., 2020), save mice from age-related memory deficits (Cree et al., 2018; Wang et al., 2020), and reverse spatial learning and memory impairment induced by isoflurane anesthesia (Li Q. et al., 2019). Based on the abovementioned studies, these two drugs, especially Clemastine, might have a potentiality to ameliorate cognitive impairment caused by anesthesia and surgery. However, there is no relevant research has been published so far. This also points out the direction for our follow-up research.

There are also some limitations in this study. First, we cannot identify the source of differentially expressed circRNAs, miRNAs, and mRNAs. Because the prefrontal cortex contains astrocytes, microglia, neurons, and other types of cells. Our sequencing results provided an overall expression trend of the differentially expressed genes. Second, NGS technology has been performed this study in aged mice, the differential expression of genes has been partly verified by qRT-PCR. It remains unclear whether the genes are differentially expressed in homo sapiens brains. Finally, we used bioinformatics analysis to predict the possible mechanisms of differentially expressed genes, and many of the mechanisms that may be related to PND need to be further verified by experiments.

## Conclusion

In summary, this study is the first one to explore the differential expression profiles of genes caused by PND. We analyzed the differentially expressed circRNAs, miRNAs, and mRNAs in the prefrontal cortex of aged mice with PND by next-generation sequencing technology and performed some validation work. Meanwhile, through bioinformatics analysis, we predicted the biological functions of differentially expressed genes, constructed a ceRNA network, provided potential targets and small molecule drugs for the treatment of PND.

## Data Availability Statement

The datasets presented in this study can be found in online repositories. The names of the repository/repositories and accession number(s) can be found below: https://www.ncbi.nlm.nih.gov/geo/, GSE174413.

## Ethics Statement

The animal study was reviewed and approved by the Institutional Animal Care and Use Committee (Approval No. SYSU-IACUC-2020-000326) and the Laboratory Animal Ethics Committee of Sun Yat-sen University.

## Author Contributions

ZW conceived and designed the study and interpreted experiments. WW and XZ performed the experiments and prepared the initial draft of the manuscript. YP, JZ, LC, W-jL, YL, and JW supervised the project. All authors read and approved the final submission.

## Conflict of Interest

The authors declare that the research was conducted in the absence of any commercial or financial relationships that could be construed as apotential conflict of interest.

## Footnotes

1 https://github.com/lh3/seqtk

2 https://github.com/lh3/bwa

3 http://circrna.org/

4 https://www.r-project.org/

5 http://hannonlab.cshl.edu/fastx_toolkit/index.html

6 https://www.perl.org/

7 https://www.string-db.org/

8 http://starbase.sysu.edu.cn/

9 http://bioinformatics.psb.ugent.be/webtools/Venn/

10 https://portals.broadinstitute.org/cmap/index.jsp
